# Lambertianic Acid Sensitizes Non-Small Cell Lung Cancers to TRAIL-Induced Apoptosis via Inhibition of XIAP/NF-κB and Activation of Caspases and Death Receptor 4

**DOI:** 10.3390/ijms19051476

**Published:** 2018-05-16

**Authors:** Deok Soo Ahn, Hyo Jung Lee, Jisung Hwang, Hyukgyu Han, Bonglee Kim, BumSang Shim, Sung-Hoon Kim

**Affiliations:** College of Korean Medicine, Kyung Hee University, Seoul 02447, Korea; crowduk@hanmail.net (D.S.A.); hyonice77@naver.com (H.J.L.); hjsung0103@naver.com (J.H.); bodyline740@nate.com (H.H.); bongleekim@khu.ac.kr (B.K.); eshimbs@khu.ac.kr (B.S.)

**Keywords:** non-small cell lung cancer, lambertianic acid, apoptosis, TRAIL, XIAP, NF-κB

## Abstract

Lambertianic acid (LA) is a biologically active compound from the leaves of *Pinus koraiensis.* In the present study, apoptotic mechanisms of LA plus TNF-related apoptosis-inducing ligand (TRAIL) were elucidated in non-small cell lung cancer cells (NSCLCs). Cytotoxicity assay, flow cytometry, immunoprecipitation, and Western blotting were performed. Here, combined treatment of LA and TRAIL increased cytotoxicity, sub-G1 population, cleaved poly (ADP-ribose) polymerase (PARP), and caspase3/8/9 in A549 and H1299 cells compared to LA or TRAIL alone. Furthermore, combined treatment of LA and TRAIL significantly decreased antiapoptotic proteins such as B-cell lymphoma 2 (Bcl-2), Fas-like inhibitor protein (FLIP), and X-linked inhibitor of apoptosis protein (XIAP), and enhanced the activation of proapoptotic proteins Bid compared to LA or TRAIL alone. In addition, combined treatment of LA and TRAIL upregulated the expression of Death receptor 4 (DR4) and downregulated phosphorylation of nuclear factor κ-light-chain-enhancer of activated B cells (p-NF-κB), inhibitory protein of kB family (p-IκB), and FLIP in A549 and H1299 cells along with disrupted binding of XIAP with caspase3 or NF-κB. Overall, these findings suggest that lambertianic acid enhances TRAIL-induced apoptosis via inhibition of XIAP/NF-κB in TRAIL resistant NSCLCs.

## 1. Introduction

The death ligand TRAIL (tumor necrosis factor-related apoptosis-inducing ligand), a member of the TNF superfamily, induces apoptosis in cancer cells with low toxicity and less resistance in normal cells [1]. TRAIL activation is mainly induced by binding to the DR4 and DR5 and subsequently leads to death-inducing signaling complexes (DISCs) via FAS-associated protein and caspase-8 protein leading to activation of apoptotic cell death [2,3]. Though TRAIL is an important anticancer agent, TRAIL resistance is a major limitation to effective cancer therapy [4,5]. Therefore, development of combination treatments to overcome resistance to TRAIL is requested for effective cancer therapy. Notably, non-small cell lung cancer cell lines such as A549, H1299, and H596 cells are known to be resistant to TRAIL-induced apoptosis [6]. Recently, several groups reported the synergistic or additive effect of phytochemicals, such as kaempferol [7], angelicin [8], and curcumin [9], on TRAIL-induced apoptosis.

**Figure 1:**
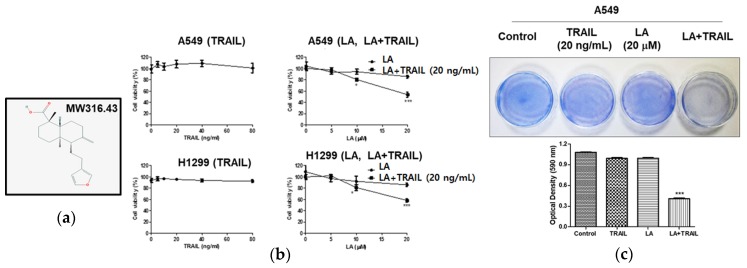
Cytotoxicity of the combination of lambertianic acid (LA) and tumor necrosis factor-related apoptosis-inducing ligand (TRAIL) in A549 and H1299 non-small cell lung cancer cells. (**a**) Chemical structure of LA. Molecular weight = 316.43. (**b**) Cell viability was evaluated by 3-(4,5-dimethylthiazol-2-yl)-2,5-diphenyltetrazolium bromide (MTT) assay. Cells were seeded onto 96 well microplates and treated with various concentrations of LA (0, 5, 10, 20 μM) and TRAIL (0, 20, 40, 80 ng/mL) for 24 h. The cytotoxic effects of LA (20 μM) and TRAIL (20 ng/mL) on TRAIL in A549 and H1299 cells. Data represent means ± SD. * *p*< 0.05, *** *p* < 0.001 versus LA-treated control (*n* = 3). (**c**) A549 cells were treated with LA (20 μM) and/or TRAIL (20 ng/mL) for 24 h and then stained with crystal violet. Data represent means ± SD. *** *p* < 0.001 versus TRAIL alone (*n* = 4).

Lambertianic acid (LA) (Figure 1a), a major active constituent of *Pinus koraiensis*, has been reported to have antiobesity [10], anti-inflammatory [11], and anticancer effects [12]. Nevertheless, the underlying apoptotic mechanism of LA as a TRAIL sensitizer has never been demonstrated. In the present study, the sensitizing mechanism of LA in TRAIL-induced apoptosis was investigated as a novel strategy to overcome the resistance of cancer cells to apoptosis in A549 and H1299 NSCLCs.

## 2. Results

### 2.1. Combined Treatment of LA and TRAIL Enhanced Cytotoxicity in A549 and H1299 Non-Small Cell Lung Cancer Cells

The cytotoxicity of LA in A549 and H1299 non-small cell lung cancer cells was evaluated by MTT assay. As shown in Figure 1b, combined treatment of LA (20 μM) and TRAIL (20 ng/mL) for 24 h showed significantly cytotoxicity in A549 and H1299 cells compared to treatment with LA or TRAIL alone (Figure 1b). Also, a cell proliferation assay using crystal violet staining revealed that combined treatment of LA (20 μM) and TRAIL (20 ng/mL) significantly inhibited proliferation in A549 cells compared to treatment with LA or TRAIL alone (Figure 1c).

### 2.2. Combined Treatment of LA and TRAIL Significantly Increased the Sub-G1 Population and Also Increased the Cleavage of PARP and Caspase8/9/3 in A549 and H1299 Non-Small Cell Lung Cancer Cells

**Figure 2:**
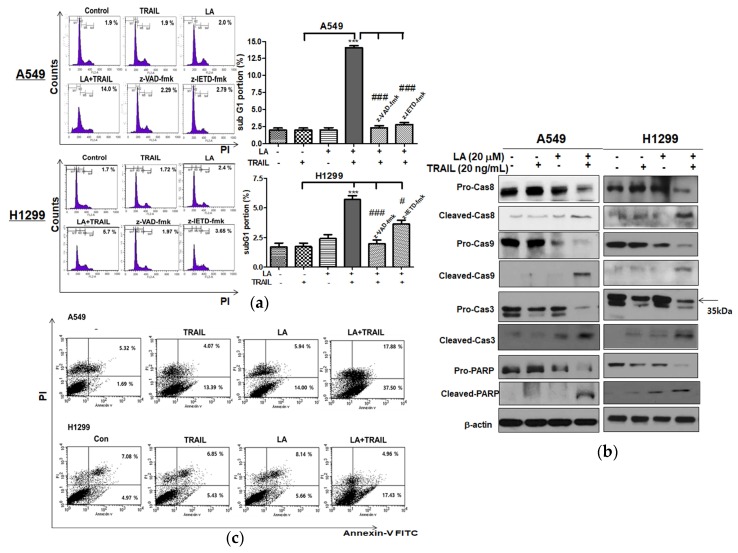
Combined effect of LA and TRAIL on the sub-G1 population and apoptotic proteins in A549 and H1299 non-small cell lung cancer cells. (**a**) Cells were treated with LA (20 μM) and/or TRAIL (20 ng/mL) for 24 h. The treated cells were fixed with 70% ethanol, stained with propidium iodide (PI) and analyzed by flow cytometry with or without with caspase inhibitors (pan caspase inhibitor; z-VAD-fmk (80 μM), caspase-8 inhibitor; z-IETD-fmk (50 μM)). Bar graphs show quantification of cell cycle population (%). Data represent means ± SD. *** *p* < 0.001 versus TRAIL alone, # *p* < 0.05, ### *p* < 0.001 versus LA+TRAIL treated control. (*n* = 3)). (**b**) Cells were treated with LA (20 μM) and/or TRAIL (20 ng/mL) for 24 h. Cell lysates were prepared and subjected to Western blotting for procaspase-8,9,3, Pro-PARP, cleaved caspase-8,9,3, and cleaved-PARP. (**c**) Cells were treated with LA (20 μM) and/or TRAIL (20 ng/mL) for 24 h. The cells were stained using FITC-Annexin V/PI dye and early and late apoptotic portions were detected by flow cytometry.

To confirm the apoptotic effect of combined treatment of LA and TRAIL, Western blot assay and cell cycle analysis were performed in A549 and H1299 cells treated by combined treatment of LA and TRAIL. Combined treatment of LA and TRAIL increased sub-G1 population in A549 and H1299 cells (Figure 2a) and also increased the cleavage of PARP and caspase8/9/3 and decreased the expression of pro-PARP and pro-caspase8/9/3 in A549 and H1299 cells compared to LA or TRAIL alone (Figure 2b). To confirm the involvement of caspases, A549 and H1299 cells were pretreated with caspase inhibitors for 1 h prior to the cotreatment. Here, pan caspase inhibitor (z-VAD-fmk) and caspase-8 inhibitor (z-IETD-fmk) significantly blocked the increase of sub-G1 population by combined treatment of LA and TRAIL (Figure 2a). Consistently, a cell apoptosis assay using Annexin-V/PI double staining revealed that combined treatment of LA (20 μM) and TRAIL (20 ng/mL) for 24 h significantly increased the early and late apoptosis to 37.50% and 17.88% in A549 cells, and 17.43% and 4.96% in H1299 cells, respectively, compared to LA (20 μM) or TRAIL (20 ng/mL) alone by Annexin V and propidium iodide (PI) staining (Figure 2c).

### 2.3. Combined Treatment of LA and TRAIL Regulated Antiapoptotic and Proapoptotic Proteins in A549 and H1299 Non-Small Cell Lung Cancer Cells

**Figure 3:**
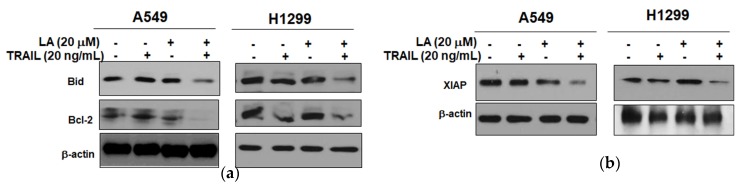
Combined effect of LA and TRAIL on antiapoptotic and proapoptotic proteins in A549 and H1299 non-small cell lung cancer cells. (**a**,**b**) Cells were treated with LA (20 µM) and/or TRAIL (20 ng/mL) for 24 h. Cell lysates were prepared and subjected to Western blotting for Bid, Bcl-2, and XIAP.

To determine whether cotreatment of LA and/or TRAIL affects apoptosis, we assessed the expression levels of proapoptotic and antiapoptotic proteins by Western blotting. Combined treatment of LA and TRAIL attenuated the expression of Bid and Bcl-2 in A549 and H1299 cells (Figure 3a). Consistently, combined treatment of LA and TRAIL effectively blocked the expression of X-linked inhibitor of apoptosis protein (XIAP) in A549 and H1299 cells compared to LA or TRAIL alone (Figure 3b).

### 2.4. Combined Treatment of LA and TRAIL Upregulated the Expression of DR4 and Inhibited the Expression of p-NF-κB, p-IκB, and FLIP in A549 and H1299 Non-Small Cell Lung Cancer Cells

**Figure 4:**
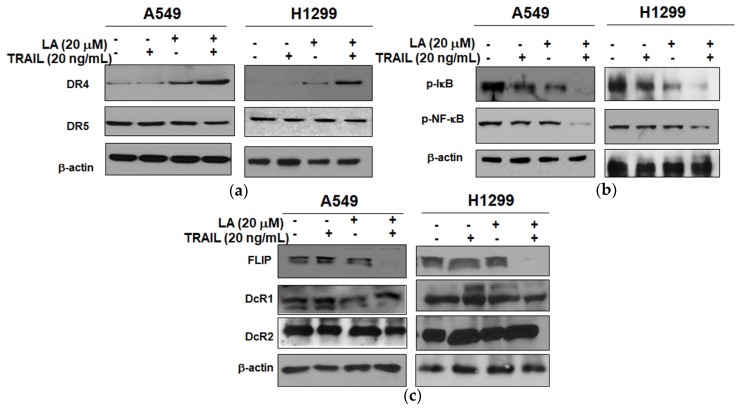
Combined effect of LA and TRAIL on the expression of DR4, p-NF-κB, p-IκB, and FLIP in A549 and H1299 cells. Cells were treated with LA (20 µM) and/or TRAIL (20 ng/mL) for 24 h and cell lysates were prepared and subjected to Western blotting. (**a**) Combined effect of LA and TRAIL on the expression of DR4 and DR5 in A549 and H1299 cells. (**b**) Combined effect of LA and TRAIL on the expression of p-NF-κB and p-IκB in A549 and H1299 cells. (**c**) Combined effect of LA and TRAIL on the expression of FLIP, DcR1, and DcR2 in A549 and H1299 cells.

We examined the expression of TRAIL death receptors and its associated proteins such as DR4 and DR5 by Western blotting. Combined treatment of LA and TRAIL upregulated the expression of DR4, but not DR5 in A549 and H1299 cells compared to LA or TRAIL alone (Figure 4a). Also, to clarify the role of NF-κB signaling in TRAIL-induced apoptosis, Western blotting was conducted in A549 and H1299 cells treated with combined treatment of LA and TRAIL. Combined treatment of LA and TRAIL attenuated the expression of p-NF-κB and p-IκB in A549 and H1299 cells compared to LA or TRAIL alone (Figure 4b). Next, the effect of TRAIL and/or LA was examined on the expression of TRAIL associated proteins such as FLIP, DcR1, and DcR2 by Western blotting. As shown in Figure 4c, combined treatment of LA (20 μM) and TRAIL (20 ng/mL) synergistically downregulated the expression of FLIP, but not DcR1 and DcR2, compared to LA or TRAIL alone.

### 2.5. Combined Treatment of LA and TRAIL Disrupted Binding of XIAP with Caspase3 and NF-κB in A549 Non-Small Cell Lung Cancer Cells

**Figure 5:**
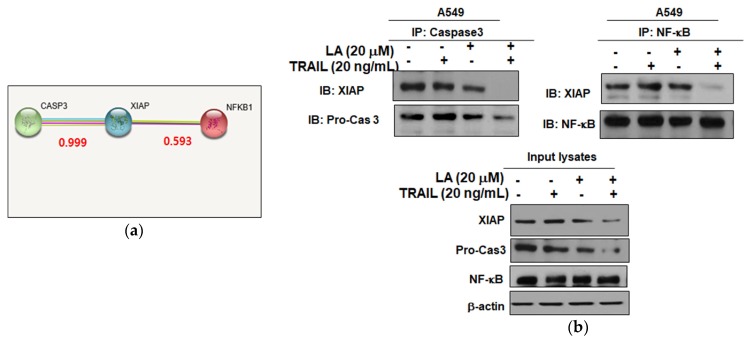
Combined effect of LA and TRAIL on XIAP interaction with caspase3 and NF-κB in A549 non-small cell lung cancer cells. (**a**) XIAP interacts with caspase3 and NF-κB in the STRING database. Red text (interaction score). (**b**) A549 cells were treated with LA (20 µM) and/or TRAIL (20 ng/mL) for 24 h. Immunoprecipitation (IP) was performed with lysates from A549 cells using anti-caspase3 and anti-NF-κB antibodies. Western blot analysis was performed to detect XIAP in whole cell lysates. Western blot analysis was performed to detect XIAP, Pro-cas3, and NF-κB in input lysates. Input lysates indicated that the 5% pre-immunoprecipitated samples and β-actin levels were confirmed as being equivalent to protein loading.

To confirm the XIAP, caspase3, and NF-κB interaction inhibition of combined treatment of LA and TRAIL, immunoprecipitation was performed in A549 cells treated with combined treatment of LA and TRAIL. Protein–protein interaction (PPI) scores of XIAP and caspase3 with NF-κB were found 0.999 and 0.593, respectively (Figure 5a). As shown in Figure 5b, the combined treatment of LA and TRAIL interrupted the binding of XIAP with Caspase3 and NF-κB.

## 3. Discussion

TRAIL is known to play an important role in apoptosis as a therapeutic agent in cancers [13,14]. Nevertheless, chemo-resistance to TRAIL has limited its clinical usage in some types of cancers [15]. To overcome this problem, combination treatment has been proposed as an attractive approach by sensitizing TRAIL-mediated cytotoxicity with less side effects [16,17]. In the current study, we investigated whether LA was able to augment TRAIL-induced apoptosis in A549 and H1299 NSCLCs that are resistance to TRAIL treatment.

Here, a combination of LA and TRAIL treatment enhanced cytotoxicity, induced sub-G1 accumulation and the cleavage of PARP, and attenuated the expression of pro-caspase8, pro-caspase9, and pro-caspase3 compared to LA or TRAIL alone in A549 and H1299 NSCLCs, implying synergistic apoptotic effect by combination of LA and TRAIL.

It was well documented that DR4 upregulation is a promising molecular target for sensitizing tumor cells to TRAIL-induced apoptosis [18]. In our study, the combination of TRAIL and LA activated DR4 in A549 and H1299 cells, implying the potent role of a death-receptor-dependent pathway by combination of TRAIL and LA.

It was reported that tumor cells acquire TRAIL resistance by the upregulation of XIAP, c-FLIP, Bcl2, and Bcl-xL as antiapoptotic proteins [19,20], and activation of phosphoinositide 3-kinase (PI3K), protein kinase B (AKT), and NF-κB as proliferation activators [21,22]. Here, the combination of LA and TRAIL attenuated the expression of Bcl-2, Bid XIAP, and FLIP in A549 and H1299 cells, implying that the combination of LA and TRAIL inhibits antiapoptotic proteins, leading to apoptosis.

There are accumulating evidences that XIAP, one of the IAP family members, contains a C-terminal RING domain and three distinct baculovirus IAP repeat (BIR) domains [23,24] and plays a critical role in NF-κB activation [25]. The BIR1/TAB1 interaction is crucial for XIAP-induced TAK1 and NF-κB (RelA/p65 and p50 subunits) activation, since the BIR2 domain of XIAP directly blocks the active sites of caspase-3 and caspase-7, while the BIR1 domain directly binds to TAB1 [25,26,27]. The NF-κB activation of XIAP is essential for cancer cell survival [25]. Thus, we examined the interaction between XIAP, caspase3, and NF-κB with a combination treatment of LA and TRAIL in A549 cells. Our results show that the combination of LA and TRAIL attenuated the expression of p-NF-κB and p-IκB and also disrupted the binding of XIAP with caspase3 or NF-κB in A549 cells, indicating that a combination of LA and TRAIL exerts an apoptotic effect via interrupted binding of XIAP with caspase3 or NF-κB.

In summary, combined treatment of LA and TRAIL increased cytotoxicity and the sub-G1 population in A549 and H1299 NSCLCs, induced apoptosis by cleavage of PARP, and inhibited pro-caspases 8/9/3, Bcl-2, and XIAP, and activated DR4 in A549 and H1299 cells. Furthermore, the combination of TRAIL and LA suppressed the expression of p-NF-κB and p-IκB. Additionally, the combination of TRAIL and LA disrupted the binding of XIAP with caspase3 or NF-κB. Taken together, our findings suggest that the combination of TRAIL and LA synergistically induces apoptosis in non-small cell lung cancer cells via the inhibition of XIAP/NF-κB as a potent TRAIL sensitizer.

## 4. Materials and Methods

### 4.1. Lambertianic Acid Isolation

*Pinus koraiensis* leaves (3 kg) were pulverized, immersed in 50% MeOH (10 L) for 3 days, and distilled to be concentrated for 10 h by using Rotary Evaporator (IKA Korea Limited, Seoul, Korea). Then, MeOH extracts were partitioned with EtOAc/distilled water (1:1) and the water layer was suspended and partitioned with n-butanol/distilled water. A part of the EtOAc fraction was subjected to celite column chromatography and eluted with CHCl_3_-MeOH (3:1) to yield 15 fractions. Among these fractions, a distinct and vivid red-purple spot from fr. 6 was isolated, purified, and identified as lambertianic acid (LA) with over 98% purity, based on spectroscopic analyses such as nuclear magnetic resonance (NMR), mass spectrometry(MS), and infrared (IR) [28].

### 4.2. Cell Culture

Human non-small cell lung cancers A549 and H1299 were obtained from American Type Culture Collection (ATCC). A549 and H1299 cells were cultured in RPMI1640 supplemented with 10% FBS and 1% antibiotic (Welgene, Gyeongsan, South Korea). 

### 4.3. Cytotoxicity Assay

The cytotoxicity of LA was measured by 3-(4,5-dimethylthiazol-2-yl)-2,5-diphenyltetrazolium bromide (MTT) assay. In brief, A549 and H1299 cells (1 × 10^4^ cells/well) were seeded onto a 96-well culture plate and exposed to various concentrations of LA for 24 h. The cells were incubated with MTT (1  mg/mL) (Sigma Chemical, St. Louis, MO, USA) for 2 h and then treated with dimethyl sulfoxide (DMSO) for 20 min. Optical density (OD) was measured using a microplate reader (Molecular Devices Co., San Jose, CA, USA) at 570 nm. Cell viability was calculated as a percentage of viable cells in LA treated group versus untreated control.

### 4.4. Crystal Violet Assay

For viability and proliferation, crystal violet assay was performed in A549 cells. The cells (2 × 10^5^ cells/well) were seeded onto a 35 mm culture plate and treated with LA (20 µM) and TRAIL (20 ng/mL) for 24 h. The cells were fixed (4% paraformaldehyde) and stained with crystal violet solution (40% ethanol, 60% PBS, and 0.5% crystal violet). Fifteen minutes later, 1 mL of 10% acetic acid was added to each well, and the absorbance was read at 590 nm using a microplate reader (Molecular Devices Co.).

### 4.5. Cell Cycle Analysis

A549 and H1299 cells (1 × 10^6^ cells/mL) were treated with LA (0, 20 µM) and TRAIL (20 ng/mL) for 24 h, washed twice with cold PBS and fixed in 75% ethanol at −20 °C. The cells were incubated with RNase A (10 mg/mL) for 1 h at 37 °C and stained with propidium iodide (50 μg/mL) for 30 min at room temperature in the dark. The stained cells were analyzed for their DNA content by FACSCalibur (Becton Dickinson, Franklin Lakes, NJ, USA) using CellQuest Software (Becton Dickinson, Franklin Lakes, NJ, USA). 

### 4.6. Western Blotting

A549 and H1299 cells (1 × 10^6^ cells/mL) were treated with various concentrations of LA for 24 h, lyzed in lysis (50 mM Tris–HCl, pH 7.4, 150 mM NaCl, 1% Triton X-100, 0.1% SDS, 1 mM EDTA, 1 mM Na_3_VO_4_, 1 mM NaF, and 1× protease inhibitor cocktail) on ice, and spun down at 14,000× *g* for 20 min at 4 °C. The supernatants were collected and quantified for protein concentration by a RC DC protein assay kit (Bio-Rad, Hercules, CA, USA). The proteins samples were separated on 4–12% NuPAGE Bis–Tris gels (Novex, Carlsbad, CA, USA) and transferred to a Hybond ECL transfer membrane for detection with antibodies for PARP Caspase-8,9,3, DR4, DR5, Bid, p-NF-κB, p-IκB, FLIP, DcR1, DcR2 (Cell Signaling Technology, Beverly, MA, USA), Bcl-2, XIAP (Santa Cruz Biotechnologies, Santa Cruz, CA, USA), and β-actin (Sigma, St. Louis, MO, USA).

### 4.7. Co-Immunoprecipitation

A549 cells were lyzed in lysis buffer (50 mM Tris–HCl, pH 7.4, 150 mM NaCl, 1% Triton X-100, 0.1% SDS, 1 mM NaF, 1 mM EDTA, 1 mM Na_3_VO_4_, and 1× protease inhibitor cocktail), and then were immunoprecipitated with caspase3 and NF-κB antibody or normal immunoglobulin G antibody. Thereafter, protein A/G sepharose beads (Santa Cruz Biotechnology, Santa Cruz, CA, USA) were applied. The final precipitated proteins were subjected to immunoblotting with the indicated antibodies.

### 4.8. Statistical Analysis

For the statistical analysis of the data, Sigmaplot version 12 software (Systat Software Inc., San Jose, CA, USA) was used. All data were expressed as means ± standard deviation (SD). A Student’s *t*-test was used for comparison of two groups. The statistically significant difference was set at *p* values of <0.05 between control and LA treated groups.

## Abbreviations

TRAIL	TNF-related apoptosis-inducing ligand
PARP	Poly (ADP-ribose) polymerase
Caspase	Cysteine aspartyl-specific protease
Bcl-2	B-cell lymphoma 2
XIAP	X-linked inhibitor of apoptosis protein
DR5	Death receptor5
NF-κB	Nuclear factor kappa-light-chain-enhancer of activated B cells
